# Myelodysplastic syndrome: the other cause of anemia in end-stage renal disease patients undergoing dialysis

**DOI:** 10.1038/s41598-020-72568-5

**Published:** 2020-09-23

**Authors:** Min-Yu Chang, Sheng-Fung Lin, Shih-Chi Wu, Wen-Chi Yang

**Affiliations:** 1 Division of Nephrology, Department of Internal Medicine, E-Da Hospital/I-Shou University, Kaohsiung, Taiwan; 2 Division of Hematology and Medical Oncology, Department of Internal Medicine, E-DA Hospital, Kaohsiung, Taiwan; 3 Trauma and Emergency Center, China Medical University Hospital, Taichung, Taiwan; 4 Graduate Institute of Clinical Medical Science, China Medical University College of Medicine, Taichung, Taiwan; 5 Faculty of School of Medicine, College of Medicine, I-Shou University, Kaohsiung, Taiwan

**Keywords:** Medical research, Risk factors

## Abstract

In end-stage renal disease (ESRD) patients receiving dialysis, anemia is common and related to a higher mortality rate. Erythropoietin (EPO) resistance and iron refractory anemia require red blood cell transfusions. Myelodysplastic syndrome (MDS) is a disease with hematopoietic dysplasia. There are limited reports regarding ESRD patients with MDS. We aim to assess whether, for ESRD patients, undergoing dialysis is a predictive factor of MDS by analyzing data from the Taiwan National Health Insurance Research Database. We enrolled 74,712 patients with chronic renal failure (ESRD) who underwent dialysis and matched 74,712 control patients. In our study, we noticed that compared with the non-ESRD controls, in ESRD patients, undergoing dialysis (subdistribution hazard ratio [sHR] = 1.60, 1.16–2.19) and age (sHR = 1.03, 1.02–1.04) had positive predictive value for MDS occurrence. Moreover, more units of red blood cell transfusion (higher than 4 units per month) was also associated with a higher incidence of MDS. The MDS cumulative incidence increased with the duration of dialysis in ESRD patients. These effects may be related to exposure to certain cytokines, including interleukin-1, tumor necrosis factor-α, and tumor growth factor-β. In conclusion, we report the novel finding that ESRD patients undergoing dialysis have an increased risk of MDS.

## Introduction

Anemia is a common feature in late-stage chronic kidney disease (CKD), especially in patients receiving dialysis. Anemia in CKD is typically normocytic, normochromic, and hypoproliferative^1^. The major mechanisms that contribute to anemia in CKD are shortened red cell survival^2^, decreased erythropoietin (EPO) production^3^, and retained inhibitors or toxic metabolites in end-stage renal disease (ESRD) that inhibit erythropoiesis^4–6^. Other recognized potential complications that impair marrow function are iron or folate deficiency^7,8^, aluminum toxicity^9,10^, and osteitis fibrosa associated with hyperparathyroidism^11^.

Myelodysplastic syndrome (MDS) is a hematological disease characterized by reduced blood cell production and dysplasia. The disease can progress from cytopenia(s) to acute myeloid leukemia (AML) through several intermediate morphological subgroups^12^. One of the characteristics of MDS is anemia, which occurs as a result of dysplastic changes and abnormalities in the bone marrow microenvironment, leading to ineffective hematopoiesis^13^. Most patients are prescribed EPO-stimulating agents, which do not have a permanent effect; therefore, subsequent blood transfusions are necessary^14^. Hamza et al*.* reported 20 hemodialysis (HD) patients with MDS, including 10 CKD patients diagnosed with MDS before HD and 10 patients diagnosed with MDS 2 years after HD. CKD patients diagnosed with MDS before HD showed lower revised international prognostic scoring system (IPSS-R) scores and better survival than those diagnosed with MDS after HD^15^. In the present large-scale study in the Taiwanese population, we investigated whether receiving dialysis increases the risk of MDS for ESRD patients.

## Results

### Patient characteristics

**Figure 1:**
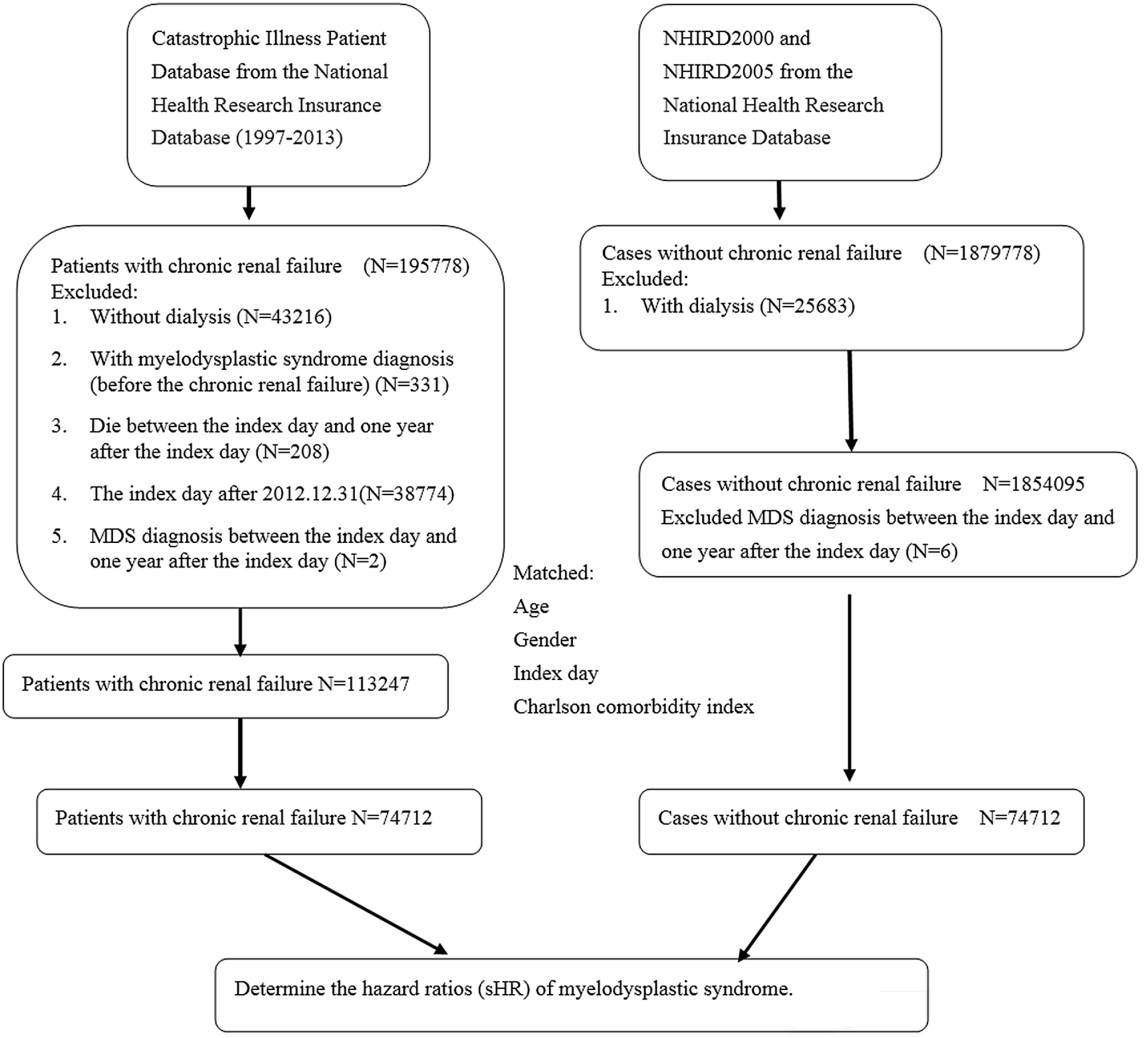
Flow chart for the enrollment criteria.

The schema of this study is shown in Fig. 1. The patients’ characteristics are shown in Table 1. Most of the patients were aged between 40 and 79 years. The control group consisted of a non-CKD population who were matched in age, sex, index day and Charlson Comorbidity Index (excluding renal function impairment) with ESRD patients undergoing dialysis. There were no differences between the ESRD and non-ESRD groups, including age and sex. However, ESRD patients had a higher incidence rate of anemia, iron-deficiency anemia (IDA) and Charlson comorbidities than the non-ESRD group, except peripheral vascular disease, chronic lung disease, diabetes, hemiplegia, cancers and acquired immune deficiency syndrome (AIDS). The incidence of MDS was similar between the ESRD and non-ESRD groups. The death rate was also higher in the ESRD group than in the non-ESRD group.

**Table 1:** Baseline characteristics of the study population.

	Non-ESRD N = 74,712	ESRD N = 74,712	*p* value
	N (%)	N (%)	
Age (y/o ± SD)*	59.55 ± 14.84	59.55 ± 14.84	0.9897
**Age group**			> .9999
< 20	545 (0.73)	545 (0.73)	
20–39	7382 (9.88)	7381 (9.88)	
40–59	27,535 (36.85)	27,536 (36.86)	
60–79	34,588 (46.3)	34,587 (46.29)	
≥ 80	4662 (6.24)	4663 (6.24)	
**Sex**			> .9999
Female	38,921 (52.09)	38,921 (52.09)	
Male	35,791 (47.91)	35,791 (47.91)	
**Area**			< .0001
North	25,688 (34.38)	23,489 (31.44)	
North Central	10,066 (13.47)	8532 (11.42)	
Central	13,019 (17.43)	13,357 (17.88)	
South Central	11,836 (15.84)	11,849 (15.86)	
Southern	11,602 (15.53)	13,656 (18.28)	
East	1981 (2.65)	1717 (2.30)	
Unknown	520 (2.65)	2112 (2.83)	
IDA	968 (1.3)	8188 (10.96)	< .0001
Anemia	2862 (3.83)	28,954 (38.75)	< .0001
**Red blood cell (unit/month)**			–
Unused		31,709 (42.44)	
< 2		27,349 (36.61)	
2–4		9566 (12.80)	
> 4		6088 (8.15)	
**Charlson comorbidities**			
Myocardial infarction	1572 (2.1)	1678 (2.25)	0.0601
Congestive heart failure	6928 (9.27)	7413 (9.92)	< .0001
Peripheral vascular disease	1769 (2.37)	1680 (2.25)	0.1252
Cerebrovascular disease	10,571 (14.15)	11,299 (15.12)	< .0001
Dementia	1254 (1.68)	1484 (1.99)	< .0001
Chronic lung disease	16,402 (21.95)	16,217 (21.71)	0.2466
Connective tissue disease	2186 (2.93)	1440 (1.93)	< .0001
Ulcer	23,519 (31.48)	22,880 (30.62)	0.0004
Chronic liver disease	10,754 (14.39)	9524 (12.75)	< .0001
Diabetes	17,649 (23.62)	17,599 (23.56)	0.7606
Diabetes with end-organ damage	3745 (5.01)	3473 (4.65)	0.0010
Hemiplegia	1404 (1.88)	1387 (1.86)	0.7453
Tumor, leukemia, lymphoma	5669 (7.59)	5616 (7.52)	0.6038
Moderate or severe liver disease	473 (0.63)	413 (0.55)	0.0432
Malignant tumor, metastasis	463 (0.62)	418 (0.56)	0.1284
AIDS	5 (0.01)	3 (0.00)	0.4795
Myelodysplastic syndrome	91 (0.12)	110 (0.15)	0.1799
Death (after 1 year)	11,730 (15.7)	42,052 (56.29)	< .0001

*Statistic method: Wilcoxon rank-sum test.

The chi-square test was used for the remaining comparisons.

### ESRD and age predict the occurrence of MDS

In our analysis, we noticed that the ESRD group had a higher occurrence rate of MDS than the non-ESRD group (subdistribution hazard ratio [sHR] = 1.60, 1.16–2.19, *p* = 0.0036*,* Table 2). Age was another predictive risk factor for MDS in the whole cohort (sHR = 1.03, 1.02–1.04, *p* < 0.001*,* Table 2). However, most of the patients in our study were between 40 and 79 years of age (Table 1). Therefore, the predictive power of age was weak and only between 40 and 79 years. We analyzed patients younger than 65 years; ESRD showed a higher prediction rate in these patients than in the whole aged population (sHR = 2.45, 1.55–3.88, *p* = 0.0001, Table 3). In patients less than 65 years, age was not a predictive factor of MDS (sHR = 1.01, 0.99–1.03, *p* = 0.5405, Table 3). Other factors, including IDA, anemia, and the Charlson Comorbidity Index (CCI), showed no predictive value for the occurrence of MDS. Only the presence of an ulcer showed a higher MDS occurrence rate in the whole cohort but not in patients younger than 65 years (Tables 2, 3).

**Table 2:** Prediction of the occurrence of myelodysplastic syndrome.

	Crude	*p* value	Adjusted	*p* value
	sHR* (95% CI)		sHR* (95% CI)	
ESRD versus Non-ESRD	1.44 (1.09–1.90)	0.0099	1.60 (1.16–2.19)	0.0036
Age	1.03 (1.02–1.04)	< .0001	1.03 (1.02–1.04)	< .0001
IDA	1.30 (0.74–2.28)	0.3617	0.94 (0.53–1.69)	0.8458
Anemia	1.49 (1.07–2.07)	0.0177	1.00 (0.69–1.46)	0.9875
**Charlson comorbidities**				
Myocardial infarction	2.69 (1.26–5.72)	0.0104	1.78 (0.82–3.84)	0.1436
Congestive heart failure	1.66 (1.04–2.64)	0.0327	0.90 (0.55–1.48)	0.6860
Peripheral vascular disease	2.08 (0.92–4.70)	0.0774	1.40 (0.61–3.18)	0.4233
Cerebrovascular disease	2.13 (1.49–3.06)	< .0001	1.30 (0.86–1.94)	0.2107
Dementia	1.60 (0.51–5.03)	0.4172	0.65 (0.20–2.10)	0.4761
Chronic lung disease	2.03 (1.48–2.79)	< .0001	1.35 (0.96–1.92)	0.0884
Connective tissue disease	2.16 (1.06–4.38)	0.0330	1.84 (0.90–3.75)	0.0947
Ulcer	1.93 (1.44–2.59)	< .0001	1.39 (1.01–1.91)	0.0459
Chronic liver disease	1.40 (0.94–2.10)	0.0986	1.16 (0.77–1.75)	0.4867
Diabetes	1.44 (1.02–2.03)	0.0382	1.00 (0.69–1.45)	0.9860
Diabetes with end-organ damage	1.43 (0.73–2.79)	0.3000	0.95 (0.46–1.93)	0.8787
Hemiplegia	2.75 (1.22–6.22)	0.0149	1.70 (0.72–4.00)	0.2264
Tumor, leukemia, lymphoma	1.69 (1.03–2.79)	0.0384	1.30 (0.78–2.16)	0.3092
Moderate or severe liver disease	NA		NA	
Malignant tumor, metastasis	NA		NA	
AIDS	NA		NA	

*Subdistribution hazard ratio.

**Table 3:** Prediction of the occurrence of myelodysplastic syndrome (age< 65).

	Crude	*p* value	Adjusted	*p* value
	sHR* (95% CI)		sHR* (95% CI)	
ESRD versus non-ESRD	2.34 (1.53–3.58)	0.0001	2.45 (1.55–3.88)	0.0001
Age	1.01 (0.99–1.03)	0.2104	1.01 (0.99–1.03)	0.5405
IDA	1.47 (0.68–3.17)	0.3293	0.99 (0.45–2.18)	0.9743
Anemia	1.50 (0.93–2.42)	0.0964	0.86 (0.51–1.46)	0.5817
**Charlson comorbidities**				
Myocardial infarction	1.51 (0.21–10.82)	0.6837	1.26 (0.17–9.17)	0.8222
Congestive heart failure	0.39 (0.05–2.79)	0.3478	0.30 (0.04–2.17)	0.2324
Peripheral vascular disease	3.43 (1.08–10.84)	0.0361	2.77 (0.86–8.90)	0.0869
Cerebrovascular disease	1.86 (0.94–3.71)	0.0766	1.14 (0.51–2.55)	0.7441
Dementia	6.48 (0.90–46.55)	0.0632	2.94 (0.38–22.78)	0.3012
Chronic lung disease	1.31 (0.73–2.36)	0.3655	1.04 (0.56–1.93)	0.9020
Connective tissue disease	1.21 (0.30–4.92)	0.7888	1.15 (0.28–4.70)	0.8451
Ulcer	1.68 (1.07–2.63)	0.0242	1.44 (0.89–2.34)	0.1390
Chronic liver disease	1.52 (0.86–2.69)	0.1483	1.30 (0.72–2.35)	0.3905
Diabetes	1.47 (0.85–2.52)	0.1667	1.20 (0.66–2.18)	0.5497
Diabetes with end-organ damage	1.64 (0.52–5.18)	0.4013	1.13 (0.33–3.82)	0.8456
Hemiplegia	4.94 (1.56–15.63)	0.0066	3.43 (0.93–12.56)	0.0631
Tumor, leukemia, lymphoma	2.64 (1.33–5.25)	0.0057	2.48 (1.23–5.00)	0.0113
Moderate or severe liver disease	NA		NA	
Malignant tumor, metastasis	NA		NA	
AIDS	NA		NA	

*Subdistribution hazard ratio.

### Transfusion dependence predicts the occurrence of MDS in ESRD patients undergoing dialysis

As shown in Table 4, ESRD patients requiring more red blood cell transfusions (more than 4 units/month; each unit contains 250 cc of whole blood) showed a higher risk of MDS occurrence (sHR = 1.96, 1.02–3.75, *p* = 0.0427, Table 4). However, this association is weak. Patients who received fewer red blood cell transfusions (less than 2 units/month and 2–4 units/month) did not show a predictive value in the development of MDS. Additionally, age was not a predictive factor for MDS development in ESRD patients.

**Table 4:** Prediction of the occurrence of myelodysplastic syndrome (ESRD patients).

	Crude	*p* value	Adjusted	*p* value
	sHR* (95% CI)		sHR* (95% CI)	
**Red blood cell (unit/month)**				
Unused	REF		REF	
< 2	0.86 (0.55–1.33)	0.4962	0.79 (0.50–1.25)	0.3165
2–4	0.97 (0.50–1.86)	0.9208	0.85 (0.43–1.69)	0.6495
> 4	2.33 (1.28–4.23)	0.0054	1.96 (1.02–3.75)	0.0427
Age	1.01 (1.00–1.03)	0.0761	1.01 (0.99–1.02)	0.2891
IDA	0.98 (0.53–1.84)	0.9577	0.89 (0.47–1.68)	0.7204
Anemia	1.03 (0.69–1.53)	0.8802	0.90 (0.58–1.38)	0.6267
**Charlson comorbidities**				
Myocardial infarction	3.46 (1.40–8.51)	0.0070	3.05 (1.21–7.72)	0.0186
Congestive heart failure	1.11 (0.54–2.28)	0.7816	0.79 (0.37–1.71)	0.5556
Peripheral vascular disease	1.90 (0.60–5.99)	0.2749	1.52 (0.48–4.88)	0.4787
Cerebrovascular disease	1.31 (0.75–2.32)	0.3445	1.03 (0.55–1.93)	0.9370
Dementia	0.78 (0.11–5.58)	0.8022	0.48 (0.06–3.56)	0.4724
Chronic lung disease	1.34 (0.84–2.15)	0.2237	1.11 (0.66–1.85)	0.6988
Connective tissue disease	2.27 (0.83–6.15)	0.1086	2.08 (0.76–5.69)	0.1557
Ulcer	1.52 (1.02–2.28)	0.0413	1.35 (0.86–2.11)	0.1954
Chronic liver disease	1.16 (0.65–2.07)	0.6170	1.03 (0.57–1.88)	0.9115
Diabetes	1.17 (0.72–1.92)	0.5276	0.98 (0.57–1.70)	0.9506
Diabetes with end-organ damage	1.22 (0.45–3.33)	0.6947	1.06 (0.36–3.07)	0.9215
Hemiplegia	1.62 (0.40–6.59)	0.4979	1.39 (0.32–6.06)	0.6589
Tumor, leukemia, lymphoma	1.58 (0.80–3.12)	0.1923	1.37 (0.68–2.77)	0.3745
Moderate or severe liver disease	NA		NA	
Malignant tumor, metastasis	NA		NA	
AIDS	NA		NA	

*Subdistribution hazard ratio.

### MDS increased with age and dialysis duration in ESRD patients

**Figure 2:**
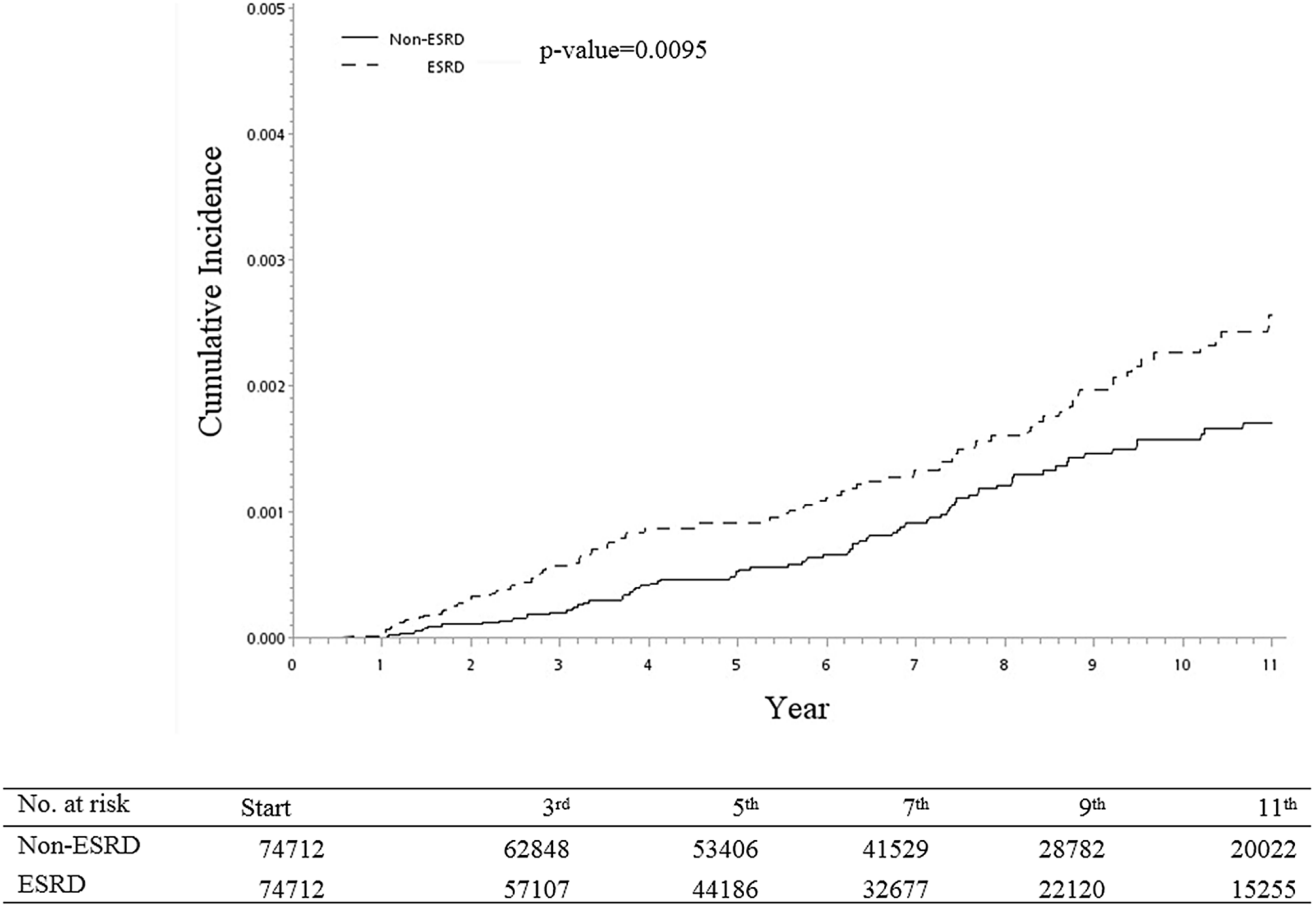
The cumulative incidences of MDS for ESRD group and matched non-ESRD groups. The cumulative incidences of MDS increases with time in both the ESRD and non-ESRD cohorts. The cumulative rate is significantly higher in the ESRD group, compared with the non-ESRD group. ESRD: end-stage renal disease; MDS: myelodysplastic syndrome.

The cumulative incidence of MDS increased with age. ESRD patients had a higher cumulative risk of MDS when they had a longer duration of dialysis. The cumulative rate was higher than that in the control population (*p* = 0.0095*,* Fig. 2).

## Discussion

Anemia is an important outcome predictor in dialysis patients. A hematocrit level of less than 20% in dialysis patients potentially increases the mortality rate to 1.5 to 3 times more than that in patients with a normal hematocrit level^16,17^. Although a relative EPO deficiency may contribute to anemia in ESRD, it is not the sole cause. Indeed, anemia in ESRD is resistant to erythropoiesis-stimulating agents (ESAs) in approximately 20–25% of patients^18^. There are some case reports assessing MDS in ESRD patients^15,19^. However, there has been no sequential relationship mentioned.

MDS is a clonal hematopoietic stem cell abnormality disorder related to genetic defects, including epigenetic pathways (DNMT3A, TET2, IDH1/2, ASXL1, EZH2, UTX), RNA splicing machinery (SF3B1, U2AF1, SRSF2, ZRSR2, PRPF8), signaling pathways (JAK2, CBL), transcription factors and corepressors (RUNX1, TP53, BCOR/BCORL1), RAS family pathways, the cohesion family, and other less frequent molecular mutations, such as SETBP1, as well as nonmolecular mechanisms, including bone marrow microenvironment factors, apoptosis, cytokines, immunoregulation, the T-cell repertoire and telomere length^20^. MDS diagnosis increases with age, and the incidence rate increases significantly after the age of 65 years^21^. Anemia with or without other cytopenia(s), including leukopenia and thrombocytopenia, is the main characteristic of MDS patients. Renal involvement in patients with MDS is rare, with a frequency from 0.48 to 4%^22–24^. Glomerular diseases associated with MDS could be membranous glomerulonephritis^23,25,26^, crescentic glomerulonephritis^22^, atheroembolic renal disease^26^, amyloidosis^22^, or mesangial proliferative glomerulonephritis with or without mesangial IgA deposition^23,24,27^. The possible mechanisms are monocytosis, increased serum and urine lysozyme levels and tumor necrosis factor-alpha (TNF-α) in MDS patients related to nephrotic syndrome or interstitial nephritis^22,25^. However, there are very limited reports showing that the incidence of MDS is increased in ESRD patients undergoing dialysis. Ayari H et al*.* reported that 10 patients with MDS, diagnosed after dialysis, had lower hemoglobin levels and higher rates of neutropenia and thrombocytopenia than the other 10 patients with MDS diagnosed before dialysis and control dialysis patients without a hematological disorder^15^.

In our cohort, we first report that in ESRD patients, treatment with dialysis for more than 6 months is an independent predicting factor for MDS based on a nationwide health insurance database. Cytokines are a possible reason for the increased MDS risk in ESRD patients undergoing dialysis. An elevation in interleukin-1 (IL-1) and TNF-α has been observed in dialysis patients^28^. Interleukin-6 (IL-6) elevation was also reported to be associated with impaired erythropoiesis and resistance to recombinant human EPO (rhEPO) in dialysis patients^29^. Transforming growth factor-β (TGF-β) is upregulated in renal disease patients and induces renal cells to produce extracellular matrix proteins leading to glomerulosclerosis, as well as tubulointerstitial fibrosis^30^. These cytokine elevations cause hypoproliferative bone marrow. A meta-analysis showed that the levels of TNF-α, IL-6, and IL-8 were significantly higher in MDS patients than in controls^31^. TNF-α is released by cytotoxic T cells and induces cell apoptosis in MDS bone marrow^20^. TGF-β, IL-6, vascular endothelial growth factor (VEGF) and interferon (IFN-α and -γ) also have myelosuppressive effects, and these cytokines have been reported to be elevated in MDS patient serum^20,32^.

The serine-threonine kinase p38 mitogen-activated protein kinase (MAPK) is known as a stress-activated kinase and has been shown to be involved in controlling the cell cycle and regulating apoptosis^33,34^. It is activated and phosphorylated in hematopoietic progenitor cells in MDS patients and is probably related to myelosuppressive cytokines (IFN-α, IFN-β, IFN-γ, TGF-β, and TNF-α)^32^. Interestingly, p38 MAPK activity is also associated with interstitial fibrosis in IgA nephropathy patients^35^ and in diabetic nephropathy^36^. The inhibition of activated p38 MAPK can improve renal function in type 2 diabetic rats^36^. We also observed that the cumulative incidence of MDS increased in patients with ESRD undergoing dialysis, along with dialysis duration, compared with the non-ESRD control group. This provides insight into whether MDS risk increases if patient exposure to certain cytokines is prolonged. However, it is still difficult to determine whether those cytokines induce the bone marrow environment changes that lead to MDS or whether both ESRD patients undergoing dialysis and MDS patients have similar cytokine profiles.

Age is a well-known factor related to MDS development. In our study, most of the population (study and matched control groups) was between 40 and 79 years old. We showed that age was a weak predictive factor in the whole cohort but not in the ESRD study group. This may be because most of the population in our cohort is of middle age to early old age. This phenomenon provides information that in ESRD patients, undergoing dialysis is a stronger risk factor for MDS development than age.

We also found that ESRD patients receiving more than 4 units of red blood cell transfusions every month had a higher MDS diagnosis rate after 1 year in our cohort. Patients may receive frequent blood transfusion due to refractory anemia caused by MDS despite ESA injections. In addition, multiple transfusions will cause the accumulation of iron to toxic levels in MDS patients^37^. Excess iron is linked to hepatic, cardiac, and endocrine damage. It is also responsible for increased progression to AML^38^. In addition, prolonged or frequent blood transfusions in MDS patients are associated with shortened leukemia-free survival (LFS) and overall survival (OS)^39^. In experimental studies, iron overload has been shown to have an inhibitory effect on hematopoiesis, affecting the function of hematopoietic stem and progenitor cells and reducing the number of hematopoietic stem cells. This could be related to the upregulated NOX4/ROS/P38 MAPK signaling pathways, suggesting that iron overload induced chronic oxidative stress in hematopoietic stem and progenitor cells^40^. Iron overload also caused stromal dysfunction in a mouse model^41^ and a change in the bone marrow microenvironment leading to clonal evolution^42^.

Hepcidin suppression is one of the mechanisms causing iron overload in MDS patients^43^. The mean hepcidin levels were consistently heterogeneous across different MDS subtypes, with the lowest levels in refractory anemia with ringed sideroblasts (RARS, 1.43 nM), which may be related to carrying a somatic mutation of *SF3B1*^44^, and the highest in refractory anemia with excess blasts (RAEB, 11.3 nM) or chronic myelomonocytic leukemia (CMML, 10.04 nM) (*p* = 0.003 by ANOVA)^45^. However, ESRD patients, who have impaired therapeutic efficacy of rhEPO and iron supply, have higher plasma hepcidin levels that inhibit iron absorption, release and recycling^46^. The role of hepcidin in ESRD is associated with anemia^47–49^, cardiovascular events^50^, and resistance to EPO^51^. Combined with our data, the inconsistent role of hepcidin in ESRD and MDS suggests that iron overload in ESRD patients who receive more units of red blood cells may be a mechanism causing the occurrence of MDS. On the other hand, MDS is a progressive disease. ESRD patients undergoing dialysis who had EPO resistance and required more red blood cell transfusions may have an earlier status of MDS.

The limitations of this study are described below. In the NHIRD, the disease was defined based on the International Classification of Diseases, Ninth Revision, Clinical Modification code. The disease code(s) of the patients were determined according to the diagnosis of specialists. Therefore, in the current study, it is hard to obtain a precise diagnosis of MDS subtypes, which may have different pathogeneses and MDS, RARS due to databank limitations, and only diagnosis codes were available. We also did not record ferritin levels in our database. However, all the insurance claims in Taiwan were scrutinized and coded by medical reimbursement specialists and peer reviewed according to the standard diagnostic criteria. If doctors or hospitals commit errors in diagnoses or coding, they will be punished with many penalties. Thus, medical staff were very concerned about the correct diagnosis codes, and we believe the codes regarding diagnoses are highly reliable. MDS is still quite rare in ESRD patients (0.15%, Table 1). Therefore, even though the data are intriguing, it is unlikely that MDS affects very many ESRD patients. However, MDS needs to be diagnosed by studying the bone marrow. The MDS diagnosis rate is probably underestimated in ESRD patients because anemia is common in ESRD patients undergoing dialysis. We cannot determine the cause and effect of transfusion demand and MDS occurrence. Further study is needed.

In conclusion, we report that ESRD patients treated with dialysis have an increased risk of MDS occurrence and that the incidence accumulates with the duration of dialysis. A high number of units of red blood cells for transfusion (more than 4 units/month) is related to MDS occurrence. However, we do not know whether more units of red blood cell transfusion are a sign or predictor of MDS. For ESRD patients who need more than four units of red blood cells per month, bone marrow studies should be considered to rule out MDS.

## Methods

### Data source

This study used data from the Taiwan National Health Insurance Research Database from 1997 to 2013. Established in 1995, the Taiwan National Health Insurance (NHI) program covers over 99% of the population and contracts 93% of the medical institutions in Taiwan^52^. Disease diagnoses were identified using the International Classification of Diseases, 9th Revision, Clinical Modification (ICD-9-CM). A disease diagnosis without valid supporting clinical findings may be considered a fraudulent claim by the NHI, with a penalty 100-fold greater than the payment claimed by the treating physician or hospital.

### Subject selection

Patients diagnosed with chronic renal failure (ICD-9 codes: 585. X) between 1997 and 2013 were identified. The date of diagnosis of patients with ESRD undergoing dialysis was considered the study index date. Patients meeting the following criteria before the index date were excluded: (1) undergoing dialysis; (2) unknown sex or birthday; (3) diagnosis of MDS; (4) index date after 2012.12.31; or (5) deceased or withdrawn from the NHI program. After the index date, patients who did not undergo dialysis, those who died within 1 year, and those who were diagnosed with MDS 1 year after the index date were excluded. Based on NHI research data, we randomly assigned index dates for subjects who had never been diagnosed with chronic renal failure and selected age-, sex-, and index year-matched subjects in our control group, using a control: case ratio of 1. We included 74,712 patients in the chronic renal failure (ESRD) with dialysis group and 74,712 in the control group (Fig. 1). We defined dialysis as patients receiving HD, peritoneal dialysis (PD) or both continuously, using the NHI treatment code, for at least six months. Finally, we included 74,712 patients in the ESRD with dialysis group and 74,712 subjects in the control group (Fig. 1). All patients were followed up from the index date to the first occurrence of one of the following: MDS, withdrawal from the NHI program, or the last day of 2013. CCI score was calculated for each subject.

### Statistical analysis

The differences in the baseline characteristics between the ESRD patient group and the control (non-ESRD) group were examined using chi-square tests for categorical variables and the Wilcoxon rank-sum test for age. To accurately assess the risk of MDS in this population, death is an important consideration. A traditional Cox survival analysis does not yield more accurate estimates. When following subjects in an observational study, follow-up until the occurrence of an event of interest is common, at which time the subjects are no longer followed. Any event of interest that could happen after the first event of interest will not be considered. Therefore, the risk of an event of interest is usually overestimated when a competing event of interest exists^53^. The aim of every study is to present accurate findings and results, which indicates the importance of considering any event that may affect the risk analysis of the main event of interest. Traditional survival analysis typically only considers one event at a time (e.g., death or MDS), possibly causing other events to be overlooked and the resulting risk estimates to be overestimated. Thus, these results should not be directly interpreted and applied in clinical settings. To overcome this issue, our study considered the competing risk of death using the Fine and Gray regression hazards model in our calculation of subdistribution hazard ratios (sHRs), a method found to be adopted by previous studies^54^, to obtain better and more accurate estimations. We adjusted HRs by other factors shown in the Tables.

Cumulative incidence curves were based on the Kaplan–Meier method, and the differences in the cumulative incidence between groups were examined using the log-rank test. All statistical analyses were performed with SAS software version 9.4 (SAS Institute Inc., Cary, NC, USA). A two-tailed *p* value below 0.05 was considered significant.

### Ethics statement

Because the NHI database entirely consists of anonymous and encrypted secondary data released to the public for research purposes, this study was exempted from a full review by the ethics review committee at the EDA hospital.

### Disclosure

This study used the National Health Insurance Research Database established by the National Health Research Institutes, with the authorization of the Bureau of National Health Insurance, Ministry of Health and Welfare of Taiwan. The interpretations and the conclusions contained herein do not represent the opinion of the aforementioned agencies and institutions. There are no financial interest in the information contained in the manuscript.
